# Unexpected high level of severe events even in low-risk profile chest pain unit patients

**DOI:** 10.1007/s00059-021-05064-9

**Published:** 2021-08-31

**Authors:** Frank Breuckmann, Stephan Settelmeier, Tienush Rassaf, Matthias Hochadel, Bernd Nowak, Thomas Voigtländer, Evangelos Giannitsis, Jochen Senges, Thomas Münzel

**Affiliations:** 1 Department of Cardiology and Vascular Medicine, West German Heart and Vascular Center Essen, University Duisburg-Essen, Essen, Germany; 2 Institute for Myocardial Infarction Research Foundation, Ludwigshafen, Germany; 3 Cardioangiologisches Centrum Bethanien, Frankfurt am Main, Germany; 4 3rd Department of Medicine, University Hospital Heidelberg, Heidelberg, Germany; 5 Department of Cardiology, University Medical Center Mainz, Johannes Gutenberg-University Mainz, Mainz, Germany

**Keywords:** Chest pain unit, Early heart attack care, Self-referral, Score, Preinfarction angina, Brustschmerzeinheit, Frühzeitige Behandlung von Herzinfarkten, Selbsteinweisung, Score, Präinfarkt-Angina

## Abstract

**Aims:**

Early heart attack awareness programs are thought to increase efficacy of chest pain units (CPU) by providing live-saving information to the community. We hypothesized that self-referral might be a feasible alternative to activation of emergency medical services (EMS) in selected chest pain patients with a specific low-risk profile.

**Methods and results:**

In this observational registry-based study, data from 4743 CPU patients were analyzed for differences between those with or without severe or fatal prehospital or in-unit events (out-of-hospital cardiac arrest and/or in-unit death, resuscitation or ventricular tachycardia). In order to identify a low-risk subset in which early self-referral might be recommended to reduce prehospital critical time intervals, the Global Registry of Acute Coronary Events (GRACE) score for in-hospital mortality and a specific low-risk CPU score developed from the data by multivariate regression analysis were applied and corresponding event rates were calculated. Male gender, cardiac symptoms other than chest pain, first onset of symptoms and a history of myocardial infarction, heart failure or cardioverter defibrillator implantation increased propensity for critical events. Event rates within the low-risk subsets varied from 0.5–2.8%. Those patients with preinfarction angina experienced fewer events.

**Conclusions:**

When educating patients and the general population about angina pectoris symptoms and early admission, activation of EMS remains recommended. Even in patients without any CPU-specific risk factor, self-referral bears the risk of severe or fatal pre- or in-unit events of 0.6%. However, admission should not be delayed, and self-referral might be feasible in patients with previous symptoms of preinfarction angina.

## Abstract (de)

**Ziele:**

Programme zur Früherkennung von Herzinfarkten sollen durch die Information der Öffentlichkeit über lebensrettende Maßnahmen die Wirksamkeit von Chest Pain Units (CPU) erhöhen. Unsere Hypothese war, dass bei ausgewählten Patienten mit Brustschmerzen und einem spezifischen niedrigen Risikoprofil die Selbsteinweisung eine praktikable Alternative zum Alarmieren des Notarztes bzw. Rettungsdienstes sein könnte**.**

**Methoden und Ergebnisse:**

In dieser registerbasierten Beobachtungsstudie wurden die Daten von 4743 CPU-Patienten dahingehend analysiert, ob es Unterschiede zwischen Patienten mit schweren oder tödlichen Ereignissen (Herzstillstand außerhalb des Krankenhauses und/oder Tod in der CPU, Wiederbelebung oder ventrikuläre Tachykardie) vor der CPU-Aufnahme oder in der CPU im Vergleich zu Patienten, bei denen dies nicht der Fall war, gibt. Um eine Untergruppe mit niedrigem Risiko zu identifizieren, bei der eine frühzeitige Selbsteinweisung empfohlen werden könnte, um kritische Zeitintervalle vor der Krankenhausaufnahme zu verkürzen, wurden der Global-Registry-of-Acute-Coronary-Events-Score (GRACE-Score) für die Mortalität im Krankenhaus und ein spezifischer CPU-Score für ein niedriges Risiko, der aus den Daten einer multivariaten Regressionsanalyse entwickelt wurde, angewandt und die korrespondierenden Ereignisraten berechnet. Männliches Geschlecht, andere kardiologische Symptome außer Brustschmerzen, erstmaliges Auftreten von Symptomen und ein vorausgegangener Herzinfarkt, Herzinsuffizienz oder die Implantation eines Kardioverter-Defibrillators erhöhten die Wahrscheinlichkeit kritischer Ereignisse. Die Ereignisraten innerhalb der Untergruppen mit niedrigem Risiko variierten von 0,5–2,8%. Patienten mit einer Präinfarkt-Angina erlebten weniger Ereignisse.

**Schlussfolgerung:**

Bei der Aufklärung von Patienten und Öffentlichkeit über die Symptome der Angina-pectoris und die Notwendigkeit zur frühzeitigen Krankenhauseinweisung wird weiterhin die Alarmierung eines Notarztes bzw. Rettungsdienstes empfohlen. Selbst bei Patienten ohne CPU-spezifische Risikofaktoren beträgt das Risiko für schwere oder tödliche Ereignisse vor oder bei Selbsteinweisung in eine CPU 0,6 %. Die Zeit bis zur stationären Aufnahme sollte weiter so kurz wie möglich gehalten werden, hier könnte lediglich bei Patienten mit vorausgegangenen Symptomen einer Präinfarkt-Angina eine Selbsteinweisung vorteilhaft sein.

## Introduction

Patients with acute severe chest pain longer than five minutes are advised to consider a myocardial infarction and accordingly to activate the emergency medical services (EMS) without any delay. Referral to a dedicated primary care facility with 24/7 catheterization lab availability is recommended [1, 2]. As of March 2020, the German Cardiac Society (DGK) had certified a total of 287 chest pain units (CPUs), thus, establishing a nationwide network across Germany [3–5]. The certification process is centrally organized by the DGK [6]. Certification is granted only for CPUs that fulfill established prerequisites. Key elements of the certification process include characteristic locations, equipment, diagnostic and therapeutic strategies, cooperation, staff education, and organization [4, 7, 8]. The efficacy in patient care as well as guideline adherence has been proven by a number of studies originating from the German CPU registry [9]. Recently, the Acute Cardiovascular Care Association also published an evidence-based framework for the development of standardized CPUs throughout Europe, which was largely adopting the German CPU certification prerequisites [10, 11]. Still, optimal integration of prehospital and hospital-based providers is crucial for timely diagnostics and reperfusion of patients with acute coronary syndromes (ACS).

More than the DGK, the American College of Cardiology Accreditation Services emphasize the role of community outreach with educational programs with the aim to identify patients with prodromal symptoms of a heart attack by an approach called early heart attack care (EHAC) [12]. Within this approach, laypersons are trained and certified to recognize early symptoms of myocardial ischemia, thereby urging chest pain patients to present to a chest pain center for further clinical rule-in or rule-out even at an early stage of disease [13]. Since tracking EHAC deputies in 2012, this program already trained more than 1 million non-healthcare persons in the United States [14]. However, the actual benefit of this program has not yet been widely evaluated and only few data are available about preclinical complications of chest pain patients.

To implement the EHAC concept and to strengthen community outreach in Germany as a potential role model for Europe, we recently initiated a grant application for a local proof-of-concept study named “proHerz” (proHeart) which is to start in 2021. This study is supported by the DGK, the German Heart Foundation and one of the major German health care insurance companies. The proHerz initiative will include the production of an online teaching platform with certification abilities for widespread training of nonprofessional caregivers [12, 15, 16].

Previously we demonstrated that patients who contact a CPU as a self-referral are younger, less severely ill and have more noncoronary problems than those calling EMS. Nevertheless, 30% of self-referral patients had an ACS [1]. Keeping in mind that the implementation of an EHAC process might lead to a further increase of self-referral, we hypothesized that it might be in particular necessary to define a dedicated chest pain patient subgroup that may not be endangered by self-referral in order to implement a new optional standard operating procedure for CPU admission accompanying the given EMS recommendations. Thus, the current study aimed to analyze whether we can identify patients with acute chest pain and low risk profile for severe or fatal prehospital or early in-unit complications that might be suitable to be advised to present as self-referrals to a CPU rather than by activation of the EMS.

## Methods

The study was designed as an observational registry-based real-world study. Data were retrieved from the German CPU registry II which served the purpose of quality assurance and scientific research and was endorsed by the DGK and the German Heart Foundation. The registry has been approved by the ethics committee of the Landesaerztekammer Rheinland-Pfalz and by the local ethical review boards. Informed consent was obtained from each patient. The study protocol conforms to the ethical guidelines of the 1975 Declaration of Helsinki. Voluntary participation in the registry was restricted to CPUs successfully certified by the DGK. The Stiftung Institut fuer Herzinfarktforschung (IHF), Ludwigshafen, was responsible for maintenance of the registry, project management and data management. A variety of parameters on preclinical and clinical basic demographic, symptom-related, diagnostic, therapeutic and outcome-related parameters were collected. Documentation was recorded via an internet-based electronic data capture system.

### Study population

For the quality assurance, consecutive all-comers admitted for chest pain to one of the participating certified CPUs were to be consecutively enrolled during a period of at least 4 weeks. Inclusion was based to an admission between 09/2015 and 08/2018. A composite of life-threatening or fatal events considered critical for self-referral of the patients was defined including out-of-hospital cardiac arrest and/or in-unit death, resuscitation or ventricular tachycardia. Incomplete documentation on those life-threatening or fatal events and/or missing informed consent served as exclusion criteria.

Patients with and without such events were compared and the following parameters were analyzed: demographics, risk factors, cardiovascular history, device therapy and selected medication. Of those, independent determinants for prehospital or in-unit life-threatening or fatal events were identified. Two separate risk scores were calculated in order to identify a low-risk group of patients admitted to the CPU.

### Low-risk CPU score favoring self-referral

For building up a specific low-risk CPU risk score for life-threatening or fatal events, clinically reasonable variables that may be known to the patient in the prehospital setting were analyzed in multivariable logistic regression models for the risk of life-threatening or fatal events. These variables included the patient baseline variables presented in Table 1. All such variables were included in a forward selection (multiple logistic regression analysis) with entry criterion of *p* < 0.1. Statistically significant variables were kept in the final score model. From the rescaled and rounded estimates of the regression coefficients, integer values were derived as contributions to the score for the significant risk factors. Thus, the low-risk CPU score was calculated as follows: implanted internal cardioverter defibrillator—3 points; previous myocardial infarction, heart failure, first onset of symptoms, syncope/presyncope—2 points each; dyspnea, arrhythmias and male gender—1 point each.

**Table 1:** Overview of patients’ demographics, including patients with and without severe or fatal prehospital or in-unit events

Demographics	Total (*n* = 4743)	Event^a^ (*n* = 125)	No event^a^ (*n* = 4618)	*p* value
Age (median, quartiles)	69 (56, 78)	70 (57, 79)	69 (56, 78)	0.45
Gender (male)	63.1%	73.6%	62.8%	*0.014*^*b*^
Smoking	26.5%	26.4%	26.5%	0.97
Arterial hypertension	71.5%	70.4%	71.5%	0.78
Hyperlipidemia	39.4%	38.4%	39.4%	0.82
Diabetes	22.5%	23.2%	22.5%	0.85
Family history for CAD	17.7%	14.4%	17.8%	0.33
Dyspnea	29.5%	29.6%	29.5%	0.98
Arrhythmias	12.1%	19.2%	11.9%	*0.013*^*b*^
Syncope	6.4%	15.2%	6.1%	*<* *0.001*^*b*^
First onset of symptoms	39.1%	54.4%	38.6%	*<* *0.001*^*b*^
Renal impairment	9.8%	16.8%	9.6%	*0.007*^*b*^
Prior MI	16.5%	23.2%	16.4%	*0.043*^*b*^
Prior PCI	26.0%	20.8%	26.2%	0.18
Prior CABG	8.3%	15.2%	8.1%	*0.005*^*b*^
Atrial fibrillation	18.4%	18.4%	18.4%	0.99
History of heart failure	8.5%	16.0%	8.3%	*0.002*^*b*^
(Dilated) cardiomyopathy	2.8%	6.4%	2.8%	*0.016*^*b*^
ICD	2.2%	7.2%	2.0%	0.076
CRT‑D	0.7%	4.8%	0.6%	*<* *0.001*^*b*^
ATT	53.1%	50.4%	53.1%	0.55
DAPT	6.8%	4.8%	6.8%	0.36
OAC	16.2%	16.0%	16.2%	0.96

*BMI* body mass index, *CAD* coronary artery disease, *MI* myocardial infarction, *PCI* percutaneous coronary intervention, *CABG* coronary artery bypass grafting, *ICD* internal cardioverter defibrillator, *CRT‑D* cardiac resynchronization therapy and defibrillator, *ATT* antithrombotic therapy, *DAPT* dual antiplatelet therapy, *OAC* oral anticoagulation

^a^Out-of-hospital cardiac arrest and/or in-unit death, resuscitation or ventricular tachycardia

^b^Significant

### Anticipated GRACE score for in-hospital mortality transferred to the prehospital situation

As at onset of symptoms in the prehospital setting only age and heart rate are known by the patients, we filled the remaining parameters with baseline parameters at the time of admission and calculated the anticipated score [17]. Risk strata were divided on the basis of the thresholds recommended in the actual CPU guidelines on ACS without persistent ST-segment elevation [7, 10].

### Statistical analysis

Demographic data and other patient characteristics are reported as percentages or absolute counts or as median with first and third quartiles. For comparisons between subgroups, the classical (Pearson’s) Chi-square test for dichotomous variables and the Wilcoxon–Mann–Whitney rank test for continuous variables were used. Results on determinants for prehospital or in-unit life-threatening or fatal events are displayed as odds ratio (OR) with 95% confidence interval (CI). *P*-values ≤ 0.05 were considered significant without adjustment for multiple testing. All *p*-values are results of two-tailed tests. The statistical computations were performed at the biometrics department of the Institute for Myocardial Infarction Research Foundation using SAS release 9.4 on a personal computer (SAS Institute, Inc., Cary, NC, USA).

## Results

A total of 4743 CPU patients fulfilled the inclusion criteria and were enrolled into the analyses. In 37.5%, ACS was named as final diagnosis, non-ardiac diagnoses were found in 19.2%. Of all 4743 patients, 2.6% experienced prehospital or in-unit life-threatening or fatal events (out-of-hospital cardiac arrest: 0.4%; in-unit death: 0.3%; resuscitation: 1.3%; ventricular tachycardia: 0.6%). Detailed patient data are given in Table 1. Whereas parameters such as family history for coronary artery disease or conventional risk factors remained without significant impact, patients with documented life-threatening or fatal events were younger, more often male, more often with a history of myocardial infarction, bypass graft placement or cardioverter defibrillator implantation and more often known to have renal impairment. In addition, patients with events more often experienced cardiac symptoms other than classical chest pain. When analyzing symptom onset, patients without life-threatening or fatal events more often experienced similar symptoms already within the days before (61.4% vs. 45.6%; *p* < 0.001).

### Independent determinants for prehospital or in-unit life-threatening or fatal events

Results are shown in Table 2. Former implantation of a cardioverter defibrillator device (with or without resynchronization), previous myocardial infarction, a history of heart failure, first onset of symptoms, syncope, dyspnea/arrhythmias, and male gender were found to favor life-threatening or fatal events. Previous percutaneous coronary intervention (PCI) was a protective factor. Age, atrial fibrillation, prior bypass graft placement, other comorbidities and cardiovascular risk factors, and antithrombotic medication were not independent predictors of the critical events.

**Table 2:** Independent determinants for prehospital or in-unit life-threatening or fatal events (c = 0.708) and their weight (1–3) for building the low-risk CPU score

Variable	OR	CI	*p* value	Weight
Gender (male)	1.71	1.12–2.63	*0.013*	1
First onset of symptoms	2.03	1.38–2.97	*<* *0.001*	2
Syncope	2.50	1.47–4.26	*0.002*	2
Dyspnea/arrhythmias	1.64	1.11–2.41	*0.013*	1
Prior MI	2.11	1.24–3.57	*0.006*	2
Heart failure	1.86	1.04–3.33	*0.036*	2
ICD/CRT‑D	3.33	1.69–6.57	*<* *0.001*	3
Prior PCI	0.44	0.25–0.76	*0.004*	–

*MI* myocardial infarction, *PCI* percutaneous coronary intervention, *ICD* internal cardioverter defibrillator, *CRT‑D* cardiac resynchronization therapy and defibrillator, *OR* odds ratio, *CI* confidence interval

### Low-risk CPU score favoring self-referral

**Fig. 1:**
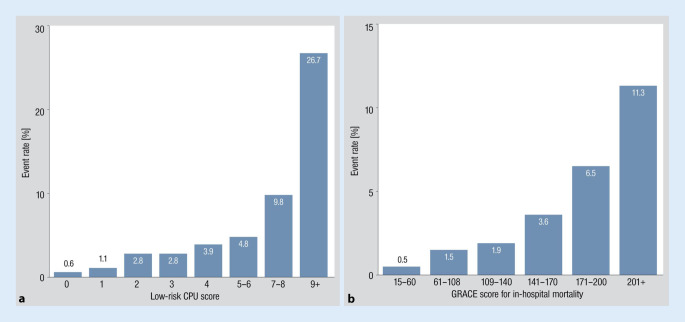
Incidence of life-threatening or fatal events in different risk strata according to the score developed from the CPU II registry (**a**) and according to the GRACE score for in-hospital mortality (**b**). Note the remaining risk of life-threatening or fatal events even within the low-risk categories

Results are displayed in Fig. 1a. In brief, 75% of patients were categorized with ≤ 3 points. The group without any risk factor comprised only 8.5% of the presenting patients. The rates of life-threatening or fatal events varied between 0.6% in the absence of any risk factor to 2.8% when scored with 3 points only. A sudden increase of events could be found between individuals with 7 to 8 points and those rated with 9 points.

### Anticipated GRACE score for in-hospital mortality transferred to the prehospital situation

Results are displayed in Fig. 1b. The event rate within the low-risk subset (GRACE scores between 15 and 108 points) varied between 0.5 and 1.5% with a sharp increase to 11.3% for individuals above 201 points. The low-risk subset represented 40.6% with the lowest risk group accounting for less than 5% of the patients.

## Discussion

With about 80%, coronary artery disease still remains the main cause for sudden cardiac death. Even though theoretically easy to treat, it is difficult to diagnose patients with significant coronary artery disease in their early stages of symptoms [12]. Usually, patients with angina or angina-like symptoms are advised to call the uniform emergency number (in Germany: 112) to activate EMS. Importantly, registry data indicate that up to one third of self-referrers are mostly low-risk patients but also patients with troponin-negative NSTE-ACS [1, 2]. Simultaneously, self-referrals show a time delay between the onsets of acute severe symptoms till their arrival to the hospital (prehospital delay) of about 4 h and about 13% present with STEMI or NSTEMI [2, 8, 18]. On the other hand, there are important arguments to encourage self-referral as a potential mode of admission that may be even expanded:

- Of all patients presenting to the CPU, 56.1% of patients were found to have non-ACS diagnoses [9].
- Some patients tend to deny and delay until further progress of symptoms in order to avoid false activation of EMS or because of mental barriers for activation of EMS [19].
- New concepts try to capture early stages of preinfarction angina by activation of bystanders, thereby addressing a higher amount of potential low-risk patients with anticipated lower risk for prehospital events [6–10].

Importantly, self-referral of patients can only be advised if it improves outcome by reducing time to treatment and if it proves to be less hazardous to the patient. CPU care is already providing optimal in-unit and in-hospital care [20]. Thus, critical time intervals within the clinics do not give room for further improvements [9, 18]. The better link into the community, e.g., via awareness campaigns may be regarded as the next escalation step of the German CPU network, aiming to capture patients in the early stages of ischemia before developing irreversible myocardial damage. The new proHerz campaign will address exactly this issue. As knowledge, awareness and education is not enough, both EHAC and proHerz encourage the involvement and training of bystanders to increase the number of active, committed caregivers beyond the level of medical professionals [9, 12, 15, 16]. The critical question to be discussed is whether this should include a new prehospital network facilitating access for individuals with preinfarction angina in order to reduce prehospital time delays and to improve acute chest pain-related mortality further. Stressing the role of self-referral as a potential alternative mode of admission in low-risk early presenting individuals was thought to be one potential component for facilitated access [1].

In our current analysis, we were unable to unequivocally identify a patient subset that might be suitable for advising self-referral without taking into account relevant risk. Even patients with zero risk according to the newly introduced low-risk CPU score exhibited a risk of 0.6% for prehospital life-threatening or fatal events and individuals with an assumed GRACE score of < 60 exhibited a corresponding prehospital event rate of 0.5%, both rapidly increasing > 1% with minimal risk criteria only. However, we could verify that those patients that developed early symptoms of ischemia days before the actual index event experienced less life-threatening or fatal events. This result suggests that either self-referral or layperson-referral through an EHAC-bystander may be a feasible alternative option of admission. If so, CPUs should increase local awareness campaigns and also offer low-threshold self-referral access. Thus, the CPU of the future will have to reach out into the community to capture patients who are at the early stages of ischemia. In line with the results of the Seattle cardiac arrest survival trial indicating impressive survival rates (62%) by increasing the number of bystander participation, benchmarking its effects at the time EHAC is launched must include information on the absence or presence of a EHAC caregiver in order to investigate if self-referral in this specific sub-subset really is feasible [12]. However, even if self-referral in low-risk patients is far more attractive than delaying symptoms of ischemia by denial, the crucial role of EMS as the key provider for preclinical care in acute chest pain patients at higher risk should be reinforced also within the proHerz concept.

### Limitations

Data from the registry are so far subject to selection bias as 22 CPUs were enrolled. As this study is a strictly observational registry, no formal test hypotheses have been specified a priori and no power calculations have been made. Therefore, the presented statistics should be interpreted in a descriptive rather than confirmatory sense. The registry collected information whether onset of symptoms was days before or shortly before presentation. However, there was no qualitative assessment of chest pain that would allow for further differentiation of preinfarction angina.

### Conclusions

Neither a specific low-risk chest pain unit (CPU) score nor the hypothetic use of the Global Registry of Acute Coronary Events (GRACE) score was able to identify of a subgroup of chest pain patients not experiencing prehospital life-threatening or fatal events. Thus, in the conventional setting as well as in the setting of early heart attack care (EHAC) with motivation for admission initiated by layperson bystanders, activation of the emergency medical services (EMS) remains the gold standard for mode of referral. However, severe events were less often seen in patients with preinfarction angina—the target population of EHAC. Thus, instead of delaying life-saving diagnostics and treatment because of too much respect to activate EMS, those patients may benefit in particular from self- or bystander-referral and an “open-CPU concept”.
